# Multiple short‐chain dehydrogenases/reductases are regulated in pathological cardiac hypertrophy

**DOI:** 10.1002/2211-5463.12506

**Published:** 2018-09-17

**Authors:** Elise Roussel, Marie‐Claude Drolet, Anne‐Marie Lavigne, Marie Arsenault, Jacques Couet

**Affiliations:** ^1^ Groupe de recherche sur les valvulopathies Centre de Recherche Institut universitaire de cardiologie et de pneumologie de Québec Université Laval Quebec City Canada

**Keywords:** aortic regurgitation, *Dhrs7c*, heart hypertrophy, short‐chain dehydrogenase/reductase

## Abstract

Cardiac hypertrophy (CH) is an important and independent predictor of morbidity and mortality. Through expression profiling, we recently identified a subset of genes (*Dhrs7c*,* Decr*,* Dhrs11*,* Dhrs4*,* Hsd11b1*,* Hsd17b10*,* Hsd17b8*,* Blvrb*,* Pecr*), all of which are members of the short‐chain dehydrogenase/reductase (SDR) superfamily and are highly expressed in the heart, that were significantly dysregulated in a rat model of CH caused by severe aortic valve regurgitation (AR). Here, we studied their expression in various models of CH, as well as factors influencing their regulation. Among the nine SDR genes studied, all but *Hsd11b1* were down‐regulated in CH models (AR rats or mice infused with either isoproterenol or angiotensin II). This regulation showed a clear sex dimorphism, being more evident in males than in females irrespective of CH levels. In neonatal rat cardiomyocytes, we observed that treatment with the α_1_‐adrenergic receptor agonist phenylephrine mostly reproduced the observations made in CH animals models. Retinoic acid, on the other hand, stimulated the expression of most of the SDR genes studied, suggesting that their expression may be related to cardiomyocyte differentiation. Indeed, levels of expression were found to be higher in the hearts of adult animals than in neonatal cardiomyocytes. In conclusion, we identified a group of genes modulated in animal models of CH and mostly in males. This could be related to the activation of the fetal gene expression program in pathological CH situations, in which these highly expressed genes are down‐regulated in the adult heart.

**Abbreviations**
11β‐HSD1: 11β‐hydroxysteroid dehydrogenase type 1
17β‐HSD8: 17β‐hydroxysteroid dehydrogenase type 8
ACEi: angiotensin I converting enzyme inhibitor
ANGII: angiotensin II
AR: aortic valve regurgitation
CH: cardiac hypertrophy
HF: heart failure
LV: left ventricle
NRCM: neonatal rat cardiomyocyte
RA: retinoic acid
SDR: short‐chain dehydrogenase/reductase

Abnormal hemodynamic overload induces cardiac hypertrophy (CH), a compensatory adaptive response aimed at maintaining cardiac output. If this adaptive response is sustained, it can lead to decompensation and heart failure (HF), an important cause of morbidity and mortality 1.

Cardiac hypertrophy is characterized by an important remodeling of the myocardial structure, a consequence of cardiomyocyte size increase and extracellular matrix rearrangement. Neurohormonal factors as well as mechanical stress cause alterations in myocardial gene expression including the reactivation of the fetal gene program 2. This feature is common to a variety of pathological conditions including ischemia, atrophy, hypoxia and diabetes in addition to hypertrophy. This return to the fetal gene program has long been considered detrimental, but others have suggested that it protects the heart against irreversible impairment and cell death.

Genes often associated with the fetal gene program include atrial and brain natriuretic peptide (*Anp* and *Bnp*), contractile protein β‐myosin heavy chain (β‐MHC or *Myh7*) and early response genes such as *c‐myc* and *c‐fos* among many others. This reactivation of the fetal gene program in the stressed heart is accompanied by the down‐regulation of the adult gene program 3.

We study a rat model of chronic volume overload caused by severe aortic valve regurgitation (AR), which is associated with a long asymptomatic period during which the left ventricle (LV) progressively dilates (eccentric remodeling) and hypertrophies 4. This process is accompanied by a decrease in LV function, occurrence of symptoms and eventually HF 5. Chronic AR, often secondary to rheumatic fever, is a condition still frequent in developing countries and in populations having less than adequate access to health care 6, 7.

Gene expression profiles have been established in several animal models of LV eccentric hypertrophy, including by us in a rat model after 2 weeks of severe AR, a period characterized by intense LV remodeling 8, 9, 10, 11. We recently reported a LV gene with an expression profile late in the disease (9 months) 12. We observed the expected activation of many genes associated with the fetal gene program both early and late in the disease. At 9 months, a general down‐regulation of genes involved in fatty acid oxidation and bioenergetics was observed. Among the cardiac genes that were modulated in our model, a significant number (26) were tagged with the molecular function of oxidoreductase activity 12.

Here, we report that many members of the short‐chain dehydrogenase/reductase (SDR) superfamily are strongly expressed in the adult heart and that their expression is reduced in the hypertrophied myocardium.

## Materials and methods

### Animal experiments

#### Rats

Wistar rats, male (350 g) and female (225–250 g), were obtained from Charles River (St Constant, QC, Canada). Pregnant rats (day 15 of gestation) were also obtained from the same provider. Severe AR was induced by retrograde puncture of the aortic valve leaflets under echocardiographic guidance as previously described 13, 14, 15. Only animals with >65% regurgitation were included in the study. A complete echo exam was performed before AR induction and at the end of the protocol as previously described 16, 17. Captopril treatment (1 g·L^−1^ in drinking water) was initiated 14 days after AR induction 17. Pregnant female rats were ordered also from Charles River and delivered at 14–15 days of gestation.

#### Mice

C57Bl6J mice (20–25 g) were also purchased from Charles River. Micro‐osmotic pumps (model 1004; Alzet, Cupertino, CA, USA), gradually releasing isoproterenol (Iso: 30 mg·kg^−1^·day^−1^), angiotensin II (AngII; 4 mg·kg^−1^·day^−1^) or vehicle (saline), were implanted subcutaneously for 14 days. The protocols were approved by the Université Laval's animal protection committee and followed the recommendations of the Canadian Council on Laboratory Animal Care.

### Cell culture

Neonatal rat cardiomyocytes (NRCMs) were prepared following an adapted protocol 18. Briefly, ventricular tissue from 1‐ to 2‐day‐old Wistar rats was subjected to enzymatic digestion in collagenase/dispase solution. Cells were then collected by low‐speed centrifugation. Enrichment of cardiomyocytes was accomplished by plating cells on culture dishes for 2 h. NRCMs were cultured in DMEM/M199 (4 : 1) supplemented with 4% horse serum, antibiotics and 1‐β‐d‐arabinofuranoside in order to prevent the growth of contaminating non‐myocytes. H9C2 cells (ATCC CRL‐1446) were cultured in DMEM supplemented with 10% fetal bovine serum.

### Micro‐array analysis

Micro‐array analysis was conducted on 9‐month‐old AR male rats as reported previously 12. Complete data (complying with the Minimum Information About a Microarray Experiment guidelines) are available at the GEO database (NCBI) under the accession number GSE17050. Genes displaying low levels of expression were discarded from the analysis. For this, we calculated the mean + 5 SD of the signals obtained from genes encoding olfactory receptors as a threshold of minimal expression to be considered as meaningful 11.

### Analysis of mRNA accumulation by quantitative RT‐PCR

The analysis of LV mRNA levels by quantitative RT‐PCR has been described in details elsewhere 11. QuantiTect^®^ and IDT (Coralville, IA, USA) primer assays (pre‐optimized specific primer pairs) and QuantiFast^®^ SYBR Green PCR kits (Qiagen, Germantown, MD, USA) were used (Table S1). We also used one pair of non‐pre‐optimized primers for (5′‐TGCAGAAAGCTGACCTATGG‐3′ and 5′‐GGGAAGAAGGTGCGGATAAA‐3′; 95 bp transcript) for the rat *Dhrs7c* gene. Cyclophilin A (*Ppia*) was used as the control ‘housekeeping’ gene.

### Western blot analysis

Crude LV homogenates were separated by SDS/PAGE. Immunoblotting was performed as described elsewhere 13, 19. All primary antibodies were used at a 1 : 1000 dilution and were purchased from Cell Signaling Technology (Danvers, MA, USA) or from Santa Cruz Biotechnology (Santa Cruz, CA, USA).

### Tubulin immunostaining

H9C2 cells were culture in Labtek chamber slides. After culture medium removal, cells were fixed in cold 4% paraformaldehyde in PBS and permeabilized with 0.1% Triton X‐100. Primary (anti‐tubulin; Sigma‐Aldrich, St Louis, MO, USA) and secondary (Alexa Fluor 488 anti‐mouse IgG, Thermo Fisher Scientific, Waltham, MA, USA) antibodies were both used at 1 : 1000 dilution. After several washes with PBS, nuclei were stained with Hoechst 33342 (1 : 10 000 in PBS). Cells were then mounted with Fluoromount G (Thermo Fisher Scientific) and observed under a Zeiss LSM 800 confocal microscope (Carl Zeiss Microscopy, Jena, Germany).

### Statistical analysis

Results are presented as the mean and standard error of the mean (SEM). Statistical significance was set at *P* < 0.05. Student's *t* test was used when two groups were compared. One‐way analysis of variance (ANOVA) was used followed by Tukey's *post hoc* test when more than two groups were compared. Data and statistical analyses were performed using prism version 7.04 for Windows (GraphPad Software, San Diego, CA, USA).

## Results

### SDR gene expression in eccentric LV hypertrophy

**Figure 1:**
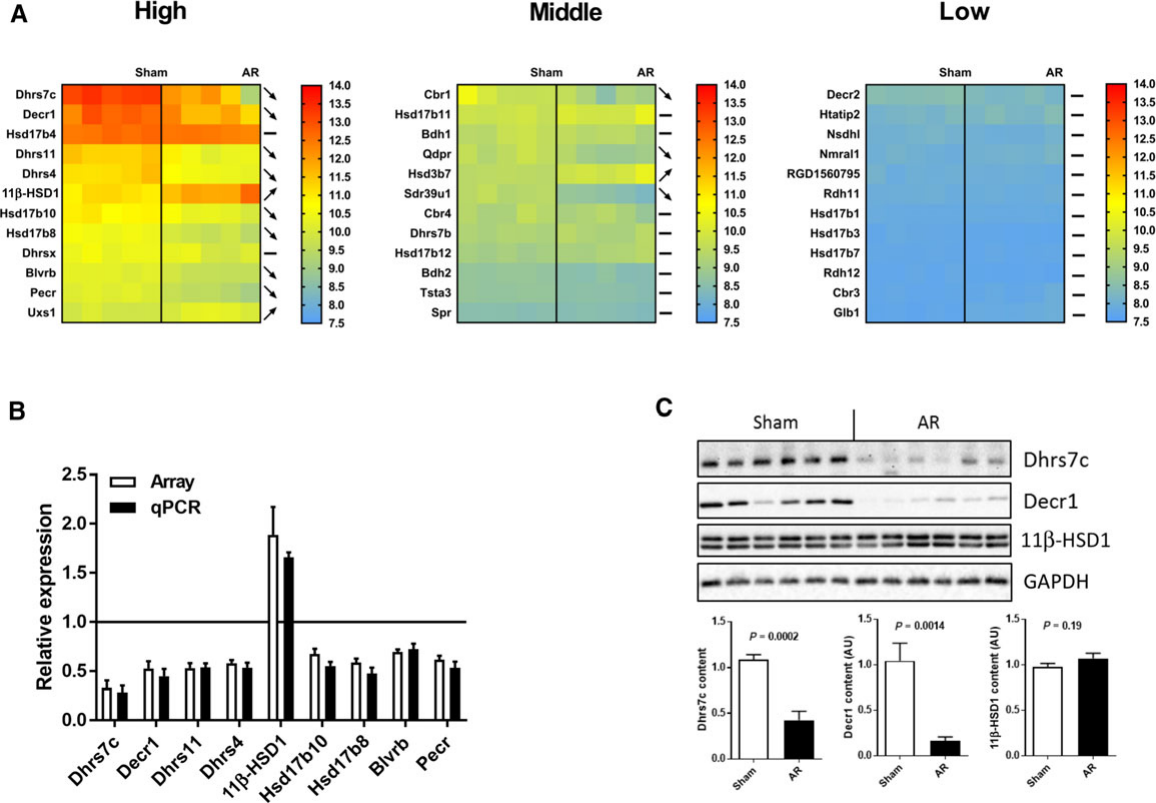
Many SDR genes highly expressed in the left ventricle (LV) of rats are down‐regulated after chronic aortic valve regurgitation (AR). (A) Heat maps of expression of 35 SDR genes in the LV of sham‐operated or AR male rats for 9 months. Genes were separated into three heat maps depending on their level of expression in sham rats. (B) Comparison of gene expression measured by micro‐array (array) technology *vs* quantitative RT‐PCR ( qPCR) for nine highly expressed SDR genes. The solid bar set at 1 represents expression of the gene in sham animals. Results are expressed as the mean ± SEM (*n* = 5). (C) Protein contents of Dhrs7c, Decr1 and 11β‐HSD1 in the myocardium of sham‐operated and AR rats. Glyceraldehyde 3‐phosphate dehydrogenase (GAPDH) protein was used as control. *P* values were determined using Student's *t* test.

After 9 months, 8 of 15 AR animals were still alive, whereas all sham‐operated animals were alive as previously reported 12. From published micro‐array data, we had identified an enriched category of genes tagged as ‘oxidoreductase’ that were down‐regulated in the myocardium of AR male rats 12. Among them, we noticed the presence of many SDR genes. In all, we identified 36 SDR genes in the rat LV displaying a higher expression signal than the fixed threshold and divided them into three groups (high, mid and low expression) as illustrated in Fig. 1A. Of these genes, 14 had significantly different levels of LV expression between AR and sham‐operated male rats. We chose to focus on nine of them (the most expressed), namely *Dhrs7c*,* Decr1*,* Dhrs11*,* Dhrs4*,* Hsd11b1*,* Hsd17b10*,* Hsd17b8*,* Blvrd* and *Pecr*. These nine genes will be referred as SRD genes from now on. With the exception of *Hsd11b1*, all of them were down‐regulated in the LV of AR rats. This was confirmed when assayed by real‐time RT‐PCR (Fig. 1B). We also observed that Dhrs7c and Decr protein contents were also strongly reduced in AR LV crude homogenates compared to shams. Only a small trend for 11β‐hydroxysteroid dehydrogenase type 1 (11β‐HSD1; encoded by *Hsd11b1*) increase was observed (Fig. 1C). We studied acute regulation of these SDR genes in the AR rat model after 48 h. Only *Dhrs7c* and *Hsd17b8* mRNA levels were down‐regulated in the hearts of AR rats 48 h post‐AR induction (Fig. S1).

### Evidence for sex dimorphism in the control of SDR genes in AR LV hypertrophy

**Figure 2:**
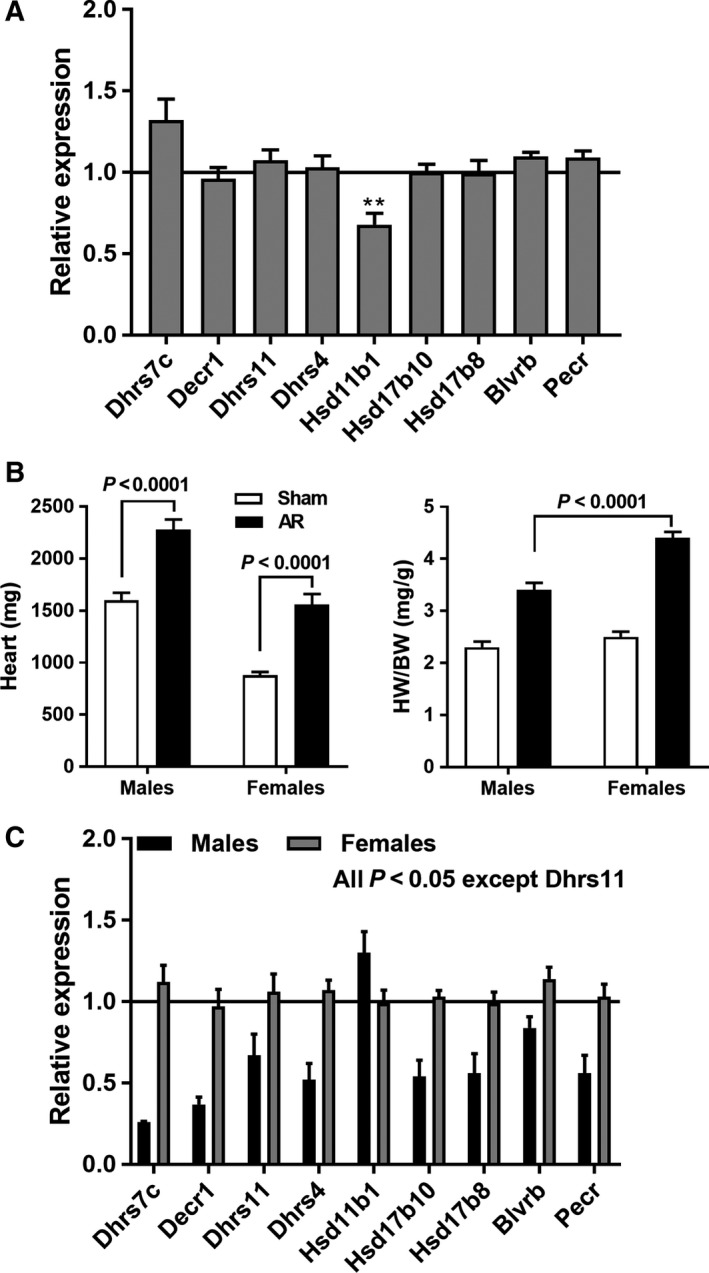
Sexual dimorphism in the regulation of SDR genes in AR rats. (A) Relative gene expression of the SDR genes in sham females compared to males. The line set at 1 represents expression of the gene in male animals. (B) Cardiac hypertrophy in male and female rats after 6 months of volume overload from AR. HW/BW: heart weight/body weight. (C) After 6 months of AR, no SDR genes are regulated in the LV of AR female rats, whereas a similar regulation of SDR genes as observed in 9‐month AR rats is observed in 6‐month AR males. Results are expressed as the mean ± SEM (*n* = 8–10 per group). Statistical significance between groups was determined using Student's *t* test.

Baseline levels of expression of the studied SDR genes were similar between male and female rats (Fig. 2A). After 6 months of severe AR, the hypertrophic response to similar levels of aortic valve regurgitation was more important in female rats (Fig. 2B). On the other hand, SDR genes remained mostly unregulated in AR females compared to males (Fig. 2C).

### SDR genes are less regulated in physiological LV remodeling

We tested two rat models of physiological heart remodeling. The first was moderate endurance training on a treadmill for a period of 6 months in both male and female Wistar rats 16. Most of the SDR genes studied were not regulated in trained rats (Fig. S2). We then tested the effects of gestation. Hearts of the dams were collected at three time points namely late pregnancy (19 days of gestation), 1 day post‐partum and 4 days post‐partum. As illustrated in Fig. S3, heart hypertrophy was present at late pregnancy and 1 day post‐partum while heart weight had returned to normal 4 days after delivery. Only mild changes in expression were observed for three of the nine genes studied, namely down‐regulation of *Dhrs7c*,* Dhrs11* and *Dhrs4*.

### Blocking LV hypertrophy development with an angiotensin I converting enzyme inhibitor tends to normalize SDR gene expression

**Figure 3:**
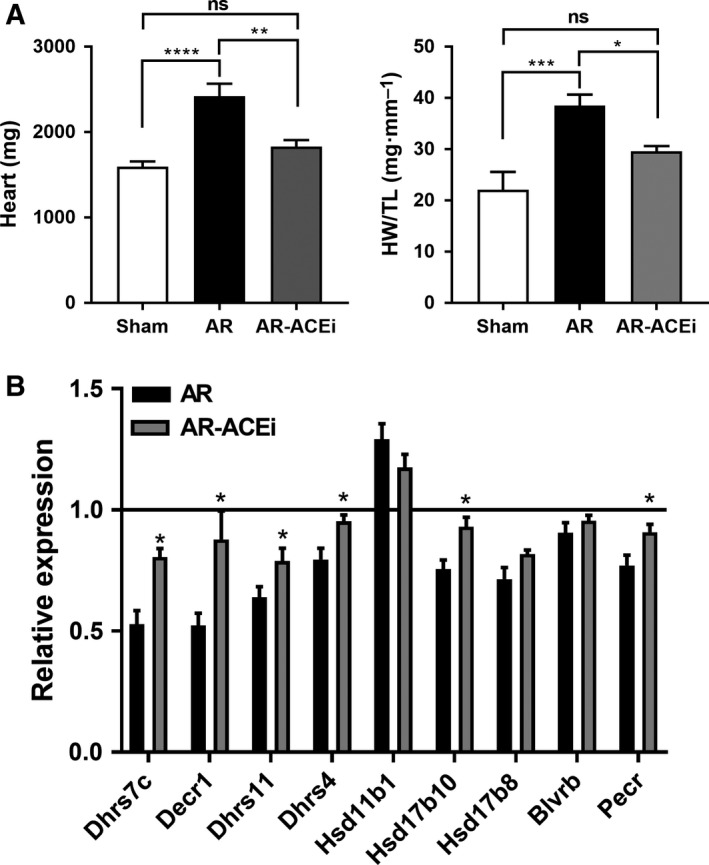
The angiotensin I converting enzyme inhibitor (ACEi) captopril reverses the hypertrophic response (A) to experimental volume overload in male AR rats and helps normalize SDR gene expression (B). Rats were treated with captopril for 6 months starting 2 weeks post‐AR induction. The line (B) set at 1 represents expression of the gene in untreated sham animals. Results are expressed as the mean ± SEM (*n* = 6 per group). (A) *P < 0.05, **P < 0.01 and ***P < 0.001 between groups; ns: non‐significant as determined using ANOVA followed by Tukey's *post hoc* test. (B) *P < 0.05 *vs* AR as determined using Student's *t* test.

Male AR rats were treated with captopril (an angiotensin I converting enzyme inhibitor; ACEi) for 6 months. This treatment mostly blocked CH development as illustrated in Fig. 3A. Six of the nine studied SDR genes had their expression normalized by the ACEi treatment (Fig. 3B).

### SDR genes are also regulated in two mouse models of concentric LV hypertrophy

**Figure 4:**
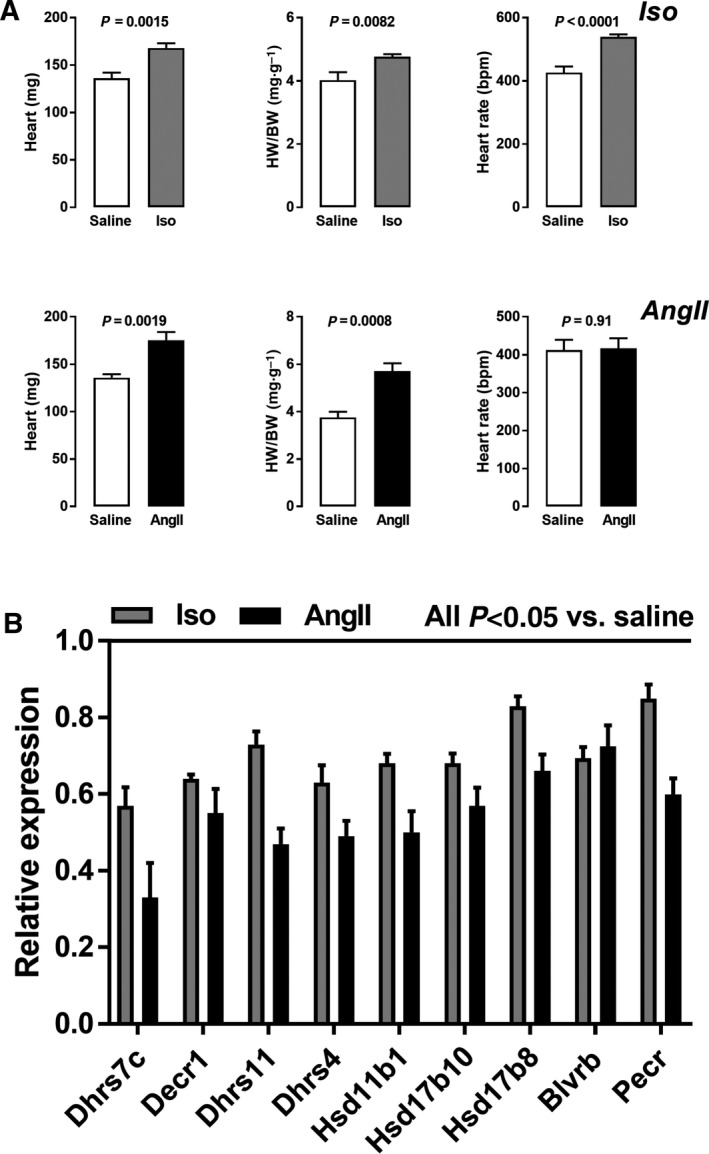
A 14‐day continuous infusion of either isoproterenol (Iso) or angiotensin II (AngII) in male mice induces mild cardiac hypertrophy (A) and general down‐regulation of SDR genes (B). Results are expressed as the mean ± SEM (*n* = 8–10 per group). Statistical significance between groups was determined using Student's *t* test.

In order to confirm that this SDR gene regulation was not limited to eccentric CH, male mice were infused continuously for 14 days with either of two pro‐hypertrophic agents, isoproterenol (Iso; β_1_‐adrenergic agonist) or angiotensin II (AngII). Both agents induced moderate levels of CH (Fig. 4A). Heart rate was significantly increased in mice receiving Iso (Fig. 4A). Echocardiography imaging confirmed the development of concentric LV hypertrophy with a significant increase in wall thickness (Tables 1 and 2). Systolic function remained unchanged for both models. The expression of all SDR genes decreased in mice infused with either Iso or AngII. This effect was stronger in mice treated with AngII (Fig. 4B). As illustrated in Figs S4 and S5, female mice receiving either Iso or AngII had a similar hypertrophic response to males. On the other hand, SDR gene regulation was less marked compared to males.

**Table 1:** Echocardiographic data after 2 weeks of continuous isoproterenol (Iso) infusion in male C57Bl6J mice. Echocardiography measurements obtained under inhaled 1.5% isoflurane anesthesia. LV, left ventricle. Relative wall thickness was calculated using the following equation: (Septal wall thickness + Posterior wall thickness)/End‐diastolic LV diameter. Values are expressed as mean ± SEM of the indicated number of animals. Statistical significance was determined using Student's *t* test

Parameters	Saline (*n* = 7)	Iso (*n* = 8)	*P*
End‐diastolic LV diameter (mm)	4.5 ± 0.07	4.5 ± 0.10	0.98
End‐systolic LV diameter (mm)	3.0 ± 0.11	2.8 ± 0.12	0.30
LV fractional shortening (%)	34 ± 2.8	38 ± 2.0	0.24
Septal wall thickness (mm)	0.68 ± 0.023	0.78 ± 0.029	0.017
Posterior wall thickness (mm)	0.68 ± 0.031	0.87 ± 0.026	0.0003
Relative wall thickness	0.30 ± 0.011	0.37 ± 0.016	0.0046

**Table 2:** Echocardiographic data after 2 weeks of continuous angiotensin II (AngII) infusion in male C57Bl6J mice. Echocardiography measurements obtained under inhaled 1.5% isoflurane anesthesia. LV, left ventricle. Relative wall thickness was calculated using the following equation: (Septal wall thickness + Posterior wall thickness)/End‐diastolic LV diameter. Values are expressed as the mean ± SEM of the indicated number of animals. Statistical significance was determined using Student's *t* test

Parameters	Saline (*n* = 6)	AngII (*n* = 7)	*P*
End‐diastolic LV diameter (mm)	4.4 ± 0.13	3.9 ± 0.07	0.013
End‐systolic LV diameter (mm)	2.9 ± 0.18	2.6 ± 0.24	0.38
LV fractional shortening (%)	34 ± 2.7	34 ± 5.3	0.99
Septal wall thickness (mm)	0.62 ± 0.024	0.74 ± 0.024	0.0039
Posterior wall thickness (mm)	0.73 ± 0.046	0.83 ± 0.035	0.099
Relative wall thickness	0.31 ± 0.012	0.40 ± 0.016	0.001

### Adrenergic agonists and retinoic acid regulate SDR genes in opposite ways *in vitro*

**Figure 5:**
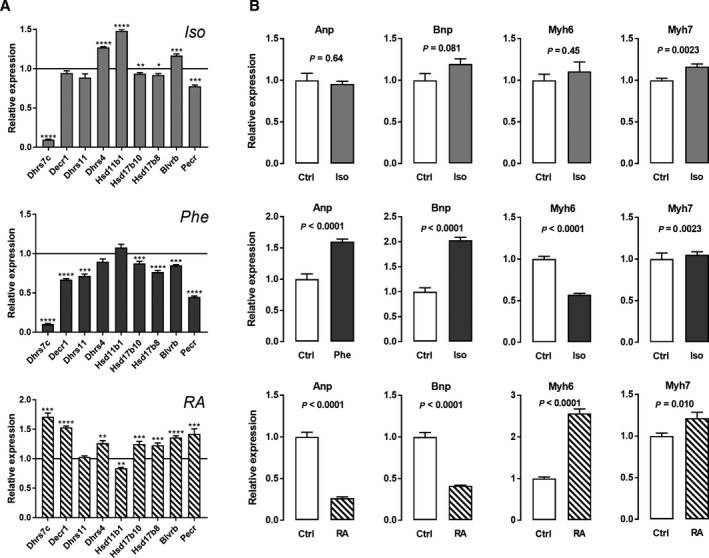
(A) Treatment of neonatal rat cardiac myocytes with Iso, Phe or all‐*trans*‐retinoic acid (RA) for 24 h regulates SDR gene expression. (B) Regulation of hypertrophy markers in cardiac myocytes by the aforementioned treatments. Results are expressed as the mean and SEM (*n* = 6 per group). *P < 0.05, **P < 0.01 and ***P < 0.001 *vs* untreated cardiac myocytes (A; bar set to 1). Statistical significance between groups was determined using Student's *t* test (A and B).

Isolated neonatal rat cardiomyocytes were treated with either 1 µm Iso, 1 µm phenylephrine (Phe; α_1_‐adrenergic agonist) or 10 nm all‐*trans*‐retinoic acid (RA) for 24 h. With the exception of the *Dhrs7c* gene, which was strongly down‐regulated by Iso and Phe, the response of the SDR genes was milder in cells treated with Iso than with Phe (Fig. 5A). RA‐treated cells showed a pattern of SDR gene expression that was a mirror image of the one observed in Phe‐treated cardiomyocytes. We also examined the expression of hypertrophy markers in these cells, namely *Anp*,* Bnp*,* Myh6* and *Myh7*. Iso treatment induced only minor changes in the expression of these genes. *Anp* and *Bnp* mRNA levels were markedly increased by Phe treatment and decreased by RA. The opposite was observed for *Myh6* (Fig. 5B).

### SDR gene expression during cardiac muscle cell differentiation

**Figure 6:**
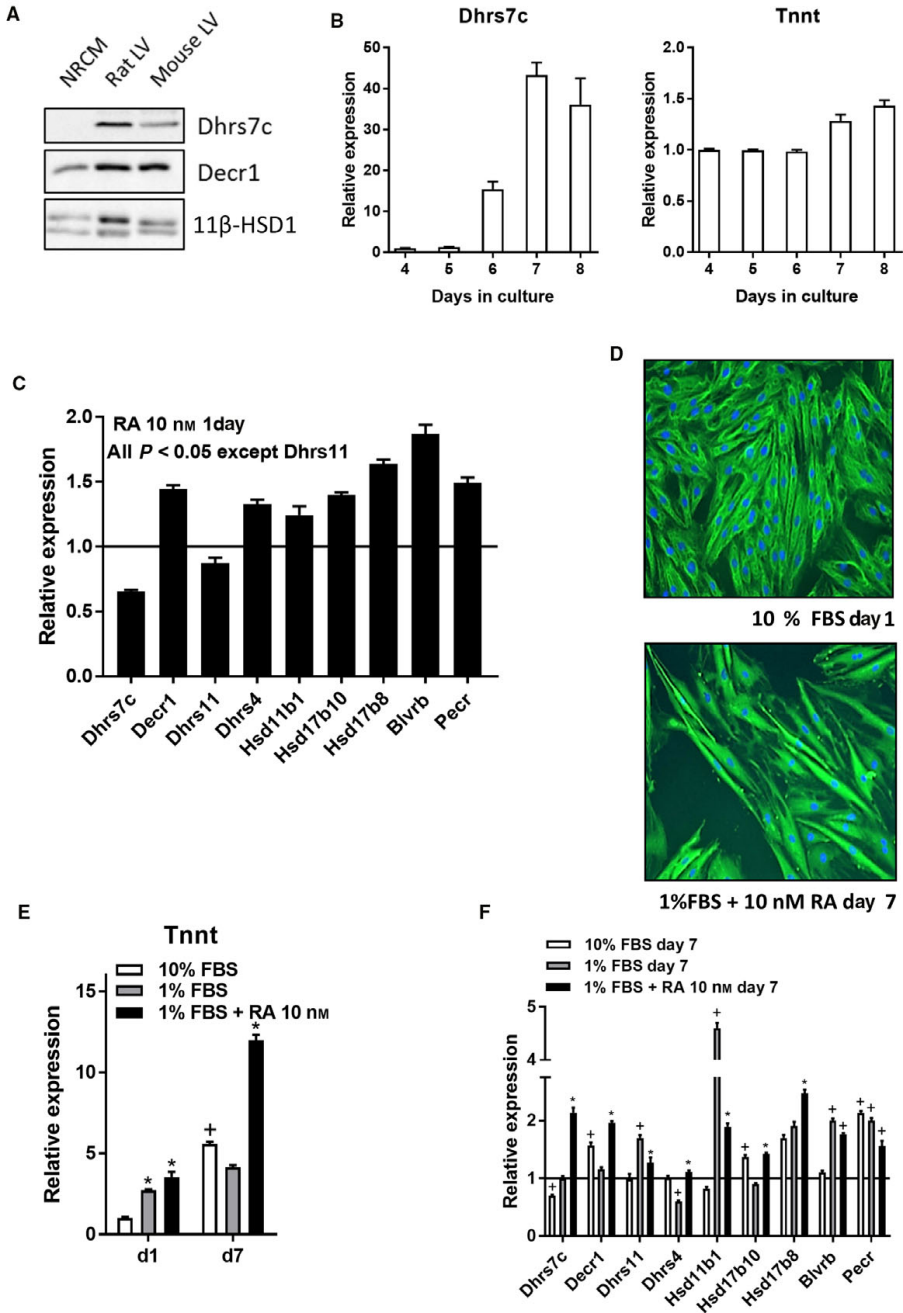
Short‐chain dehydrogenase/reductase gene expression and protein content are up‐regulated upon cardiac myocytes differentiation. (A) Dhrs7c, Decr1 or 11β‐HSD1 protein content is more abundant in adult rat or mouse LV tissue than in neonatal rat cardiac myocytes (NRCMs). (B) *Dhrs7c* and *Decr1* mRNA levels are up‐regulated with time in culture in NRCMs. (C) Treatment of H9C2 cardiac myoblasts with RA for 24 h up‐regulates SDR gene expression. (D,E) Treatment with differentiation culture medium (1% serum (FBS) + RA 10 nm) results in H9C2 morphological changes (magnification: X200) (D) and strong induction of troponin t (*Tnnt*) gene expression (E). (F) Most SDR genes are up‐regulated upon H9C2 cardiac myoblast differentiation (7 days). ^+^ *P* < 0.05 vs H9C2 cells after 24 h of culture in normal medium (10% FBS); *P < 0.05 *vs* cells after 7 days of culture in 1% FBS. Results are expressed as the mean ± SEM (*n* = 6 per group). Statistical significance between groups was determined using Student's *t* test (C) or ANOVA and Tukey's *post hoc* test (E,F).

As illustrated in Fig. 6A, Dhrs7c, Decr and 11β‐HSD1 protein levels are higher in LV crude homogenates (rat or mouse) than in NRCMs (same amounts of total protein). Expression of the *Dhrs7c* gene increased with time in NRCMs. The same was true for *Decr1* (not shown). Expression of troponin T (*Tnnt*), a marker of differentiation for cardiomyocytes also goes up (Fig. 6A). We then studied H9C2 cells. After 24 h of RA treatment (10 nm), mRNA levels of seven out of nine SDR genes studied were increased compared to untreated H9C2 cells (Fig. 6B). This cardiac myoblast cell line can be made to differentiate into cardiomyocytes upon treatment with all‐*trans*‐retinoic acid (RA) and reduction of serum concentration in culture medium. After 7 days, the differentiation culture medium (1% serum + 10 nm RA) induced formation of myotubes as illustrated in Fig. 6C. This was accompanied by a spike of expression of *Tnnt*. After 7 days of this treatment with RA, *Tnnt* expression was up by more than 12 times, which was not the case for cells cultured without RA (Fig. 6D). This differentiation process leads to an increase in expression of several SDR genes, namely *Dhrs7c*,* Decr1*,* Dhrs4* and *Hsd17b8*.

## Discussion

The results presented herein indicate that expression of highly expressed cardiac SDR genes is regulated in the stressed heart. Furthermore, this regulation is mostly present in males and less so in females. SDR genes are modulated strongly in pathological heart hypertrophy. In physiological situations leading to myocardial remodeling such as exercise and gestation, this modulation was only present for a few genes. Cardiac SDR gene expression seems to be increased during heart maturation and/or cardiac myoblast differentiation into cardiac myotubes suggesting that these genes are part of the adult gene program.

The SDR superfamily represents one of the largest and oldest protein families 20, 21, 22. They share low sequence identity but are relatively the same size and usually catalyze NAD(P)(H)‐dependent reduction/oxidation. Their large spectrum of substrates includes retinoids, steroids, polyols, fatty acid derivatives, prostaglandins and xenobiotics 20. Over 75 human SDR proteins have been identified. If enzymes of this superfamily involved in the biosynthesis of retinoids, sex hormones and glucocorticoids have received considerable attention, 30% of all SDRs are not characterized and their cellular functions remain undetermined.

Dhrs7c is mainly expressed in cardiac and skeletal muscles and is localized in the endoplasmic/sarcoplasmic reticulum 23, 24. It is predicted to have NAD/NADH activity but its function is still debated. It was first proposed to be a retinol dehydrogenase but this has been put into question in a recent study 24, 25. A role for Dhrs7c in maintaining intracellular Ca^2+^ homeostasis in the myoblastic C2C12 cell line has been proposed. Loss of Dhrs7c function leads to myotube enlargement. Dhrs7C deficiency, on the other hand, was associated with higher resting Ca^2+^ cytosolic concentrations and general Ca^2+^ overload in C2C12 skeletal muscle cells, a feature also relevant to cardiac hypertrophy 25. The NAD/NADH‐dependent dehydrogenase activity was also shown to be essential for Dhrs7C action on calcium control. More recently, in transgenic mice overexpressing Dhrs7C in skeletal muscle, a role for this SDR was identified as an enhancer of glucose metabolism and muscle performance. Dhrs7C was shown to increase glucose uptake into muscles. The role of Dhrs7c would be mediated via its retinol dehydrogenase activity and increased synthesis of RA. RA is an enhancer of insulin signaling, which results in increased plasma membrane localization of glucose transporter 4 26. Here, we demonstrate that Dhrs7c was down‐regulated in various models of CH. This had been also reported in HF in humans as well as in HF animal models 23, 27. Interestingly, we showed that female AR rats did not modulate their Dhrs7C levels as well as most of the SDRs studied here. Cardiac myocyte calcium level handling is different between the sexes and is usually better maintained in the failing heart of females 28. The reasons and the consequences of the disappearance of Dhrs7C in CH and HF at least in males have to be better explored in the future.

A metabolic link to gene expression in the stressed heart has been proposed 2. The fetal heart relies mostly on carbohydrates for energy production. After birth, this state rapidly switches to the oxidation of fatty acids. The opposite happens in various heart pathological conditions with the return to the fetal gene program and an increased reliance again on carbohydrates for energy production 29. *Dhrs7c* and *Decr1* mRNA levels increased in the adult heart compared to neonatal rat cardiomyocytes. RA, a pro‐differentiation factor, stimulates the expression of almost all SDR genes studied with the exception of *Hsd11b1*, the only gene initially observed to be up‐regulated in CH. *Decr1*,* Pecr* and *Hsd17b10* genes all encode enzymes implicated in fatty acid metabolism. Decr1 and Pecr are both 2,4‐dienoyl‐CoA reductases 30, 31. Decr1 is expressed in the mitochondria and is an auxiliary enzyme implicated in the metabolism of poly‐unsaturated fatty acids. Pecr fulfils the same function as Decr1 in the peroxisomal compartment. Unlike Decr1, Pecr is able to metabolize very long‐chain fatty acids (C > 20). *Hsd17b10* encodes a mitochondrial form of 17β‐hydroxysteroid dehydrogenase 32. Also named short‐chain l‐3‐hydroxyacyl‐CoA dehydrogenase, it is essential for the metabolism of branched‐chain and straight chain fatty acids as well as isoleucine. Other roles for this enzyme include inactivation of 17β‐estradiol as well as conversion of other steroids via a 3α‐hydroxysteroid dehydrogenase activity. We recently showed that expression of virtually all genes implicated in fatty acid oxidation was down‐regulated in male rats with severe AR but not females, including *Decr1* 33. Here, we show that *Pecr* and *Hsd17b10* also follow this trend.

*Hsd11b1* encodes 11β‐HSD1. This enzyme catalyzes the regeneration of active glucocorticoids such as cortisone or corticosterone from their 11‐keto inactive forms. Its inhibition was shown to be able to reverse established hypertrophy in a mouse model of perfusion deficit‐induced cardiac remodeling 34. Progression towards HF is slowed in mice lacking 11β‐HSD1 in a myocardial infarct model 35, 36. Of the highly expressed SDR genes studied, *Hsd11b1* was the only one to see its expression increased in the AR rat. This was also the case in Iso‐treated neonatal rat cardiomyocytes. However, in mice in which cardiac hypertrophy was induced by infusion of either Iso or AngII, *Hsd11b1* expression was reduced. In the Iso‐ or AngII‐induced hypertrophy mice, *Hsd11b1* expression was reduced, however. It is not clear which myocardial cell type (endothelial cells, cardiomyocytes or fibroblasts) is responsible for 11β‐HSD1 action in the heart 37.

Two other Dhrs (4 and 11) were found down‐regulated in CH. The putative roles of these enzymes in the heart remain obscure. *Dhrs4* encodes a carbonyl reductase with a dual function in the metabolism of endogenous signaling molecules and the detoxification of exogenous carbonyl compounds. Although, highly expressed in the heart, its physiological role remains mostly unexplored 38. On the other hand, the *Dhrs11* gene encodes a recently characterized 17β‐hydroxysteroid dehydrogenase with unusual secondary 3‐ketosteroid reductase activity 39. The *HSD17b8* gene encodes 17β‐hydroxysteroid dehydrogenase type 8 (17β‐HSD8), which has been shown to catalyze steroid oxidation/reduction *in vitro* 40. Interestingly, 17β‐HSD8 can form a heterotetramer with the carbonyl reductase type 4 to generate a mitochondrial enzyme implicated in fatty acid synthesis 41. Again, the role of 17β‐HSD8 has not been studied.

Blvrb or biliverdin reductase has been identified as a multifunctional enzyme. One of its functions is the reduction of biliverdin to bilirubin. The other functions of Blvrb include a dual‐specificity kinase activity, it being a transcription factor and molecular scaffold and cellular transporter of kinases and regulatory factors 42. Biliverdin reductase in addition to heme oxidases is part of the defense against cytotoxicity in cardiac myocytes 43.

Short‐chain dehydrogenases/reductases catalyze oxidation/reduction reactions using NAD(H)/NADP(H) as cofactors. Control of NAD homeostasis is central to heart energy metabolism. A broad range of activities related to cell signaling, Ca^2+^ handling, reactive oxygen species detoxification and others also relies on the NAD^+^ family of cofactors. NAD^+^ levels go down in the ageing heart and in HF 44, 45. The decrease described here in the expression of various SDR genes encoding enzymes using NAD(H)/NADP(H) may be a strategy used by the myocardium to focus the use of these cofactors on their main task, producing enough energy to maintain contraction. The return the fetal gene program has been proposed in the past as a way to protect the heart against stresses such as hypoxia, ischemia, hypertrophy or atrophy 3. In the AR‐dilated LV, we had already observed a decrease in the capillary density suggesting that access to oxygen and nutrients may be impaired 12, 46. The myocardium thus has to rely less on aerobic metabolism and more on glycolysis. This study relied mainly on evaluation of gene expression levels, and more thorough analyses at the level of protein content, activity and localization are needed.

## Conclusion

In this study, we identified a group of genes that are members of the SDR superfamily highly expressed in the heart muscle and regulated in response to pathological hypertrophic stresses. Many of these SDR genes seem to be part of the adult gene program that becomes inhibited when the fetal cardiac gene program is reactivated during cardiac hypertrophy and HF.

## Author contributions

ER performed most *in vitro* studies, and compiled and analyzed data. MCD performed animal studies, and compiled and analyzed data. AML performed studies in Fig. 6. MA helped in conceiving the study and writhing the manuscript. JC conceived the study and wrote the manuscript.

## Supporting information


**Fig. S1.** Evaluation by real‐time quantitative RT‐PCR of the mRNA levels of SDR genes in the LV of rats with acute and severe AR (2 days). Results are reported in arbitrary units (AU) as mean ± SEM (*n* = 5–6 per group). Levels in sham animals were fixed to 1 (line). *P < 0.05 *vs* sham. Statistical significance was determined using Student's *t* test.
**Fig. S2.** Impact of moderate intensity training for a period of 3 months on SDR gene expression in male and female rat cardiac tissue. Results are reported in arbitrary units (AU) as mean ± SEM (*n* = 5–6 per group). *P < 0.05 *vs* sedentary rats of the corresponding sex (line at 1). Statistical significance was determined using Student's *t* test.
**Fig. S3.** Impact of gestation on SDR gene expression in female mouse cardiac tissue. Top, heart weight; bottom, SDR gene expression in female rat LV. Results are reported as mean ± SEM (*n* = 4–8 per group). mRNA levels of non‐pregnant rats (NP) were normalized to 1 (line). *P < 0.05 and ****P < 0.0001 *vs* NP rats. LP, late pregnancy (19 days of gestation); 1PP (24 h post‐partum); 4PP, 3–5 days post‐partum. Statistical significance was determined using Student's *t* test.
**Fig. S4.** Impact of pro‐hypertrophic factors on SDR gene expression in female mouse cardiac tissue. (A) SDR gene expression is lower in female mouse LV compared to males. (B) Isoproterenol (Iso; gray bars) or angiotensin II (AngII; black bars) continuous infusion for 14 days results in moderate heart hypertrophy in female mice. (C) Evaluation by real‐time quantitative RT‐PCR of the LV mRNA levels of the studied SDR genes in female mice. Results are reported in arbitrary units (AU) as mean ± SEM (*n* = 5–6 per group). mRNA levels of mice receiving vehicle (saline) were normalized to 1 (line). *P < 0.05 *vs* vehicle‐treated mice. Statistical significance was determined using Student's *t* test.
**Fig. S5.** Echocardiographic data from female mice treated with continuous infusion of either isoproterenol (Iso) or angiotensin II (AngII) for 14 days. Results are reported as mean ± SEM (*n* = 5–6 per group). Statistical significance was determined using Student's *t* test.
**Table S1.** Primer assays used in qPCR analysis of gene expression.
