# Supplementary material for: Multiple short‐chain dehydrogenases/reductases are regulated in pathological cardiac hypertrophy
Source: FEBS Open Bio. 2018 Sep 17;8(10):1624–35. doi: 10.1002/2211-5463.12506 (PMC6168690; doi:10.1002/2211-5463.12506)
Supplement: Supplementary file 1 — Fig. S1. Evaluation by real‐time quantitative RT‐PCR of the mRNA levels of SDR genes in the LV of rats with acute and severe AR (2 days). Results are reported in arbitrary units (AU) as mean ± SEM (n = 5–6 per group). Levels in sham animals were fixed to 1 (line). *P < 0.05 vs sham. Statistical significance was determined using Student's t test. Fig. S2. Impact of moderate intensity training for a period of 3 months on SDR gene expression in male and female rat cardiac tissue. Results are reported in arbitrary units (AU) as mean ± SEM (n = 5–6 per group). *P < 0.05 vs sedentary rats of the corresponding sex (line at 1). Statistical significance was determined using Student's t test. Fig. S3. Impact of gestation on SDR gene expression in female mouse cardiac tissue. Top, heart weight; bottom, SDR gene expression in female rat LV. Results are reported as mean ± SEM (n = 4–8 per group). mRNA levels of non‐pregnant rats (NP) were normalized to 1 (line). *P < 0.05 and ****P < 0.0001 vs NP rats. LP, late pregnancy (19 days of gestation); 1PP (24 h post‐partum); 4PP, 3–5 days post‐partum. Statistical significance was determined using Student's t test. Fig. S4. Impact of pro‐hypertrophic factors on SDR gene expression in female mouse cardiac tissue. (A) SDR gene expression is lower in female mouse LV compared to males. (B) Isoproterenol (Iso; gray bars) or angiotensin II (AngII; black bars) continuous infusion for 14 days results in moderate heart hypertrophy in female mice. (C) Evaluation by real‐time quantitative RT‐PCR of the LV mRNA levels of the studied SDR genes in female mice. Results are reported in arbitrary units (AU) as mean ± SEM (n = 5–6 per group). mRNA levels of mice receiving vehicle (saline) were normalized to 1 (line). *P < 0.05 vs vehicle‐treated mice. Statistical significance was determined using Student's t test. Fig. S5. Echocardiographic data from female mice treated with continuous infusion of either isoproterenol (Iso) or angiotensin II (AngII) for [file FEB4-8-1624-s001.docx]

Multiple short-chain dehydrogenases/reductases are regulated in cardiac pathological hypertrophy.

Short title: SDRs in cardiac hypertrophy.

Elise Roussel, Marie-Claude Drolet, Anne-Marie Lavigne, Marie Arsenault and Jacques Couet.

Groupe de recherche sur les valvulopathies, Centre de Recherche, Institut universitaire de cardiologie et de pneumologie de Québec, Université Laval, Quebec City, Canada.

Corresponding authors: Jacques Couet PhD and Marie Arsenault MD

Groupe de Recherche en Valvulopathies, Centre de Recherche,

Institut universitaire de cardiologie et de pneumologie de Québec

2725, Chemin Sainte-Foy, Québec, (Québec), Canada, G1V 4G5

Phone: 1-418-656-4760; Fax: 1-418-656-4509

Email: [jacques.couet@med.ulaval.ca](mailto:jacques.couet@med.ulaval.ca) or [marie.arsenault@criucpq.ulaval.ca](mailto:marie.arsenault@criucpq.ulaval.ca)

**Table S1.** Primer Assays used in qPCR analysis of gene expression.

| mRNA | Species | Symbol | Cat. No. | Amplicon (bp) |
| --- | --- | --- | --- | --- |
| Biliverdin reductase B | Rat | Blvrb | Rn.PT.58.10939309 | 122 |
| Biliverdin reductase B | Mouse | Blvrb | Mm.PT.56a.30905795 | 118 |
| 2,4-dienoyl CoA reductase 1, mitochondrial | Rat | Decr1 | Rn.PT.58.44352482 | 120 |
| 2,4-dienoyl CoA reductase 1, mitochondrial | Mouse | Decr1 | Mm.PT.58.6964635 | 135 |
| 2,4-dienoyl CoA reductase 1, peroxisome | Rat | Pecr | Rn.PT.58.44811178 | 134 |
| 2,4-dienoyl CoA reductase 1, peroxisome | Mouse | Pecr | Mm.PT.58.31716864 | 129 |
| Cyclophilin a | Rat | Ppia | QT00177394 | 106 |
| Cyclophilin a | Mouse | Ppia | Mm.PT.39a.2.gs | 85 |
| dehydrogenase/reductase (SDR family) member 4 | Rat | Dhrs4 | Rn.PT.58.35669167 | 119 |
| dehydrogenase/reductase (SDR family) member 4 | Mouse | Dhrs4 | Mm.PT.58.33332410 | 116 |
| dehydrogenase/reductase (SDR family) member 7c | Rat | Dhrs7c | Non-optimized primers | 95 |
| dehydrogenase/reductase (SDR family) member 7c | Mouse | Dhrs7c | Mm.PT.58.6664141 | 92 |
| dehydrogenase/reductase (SDR family) member 11 | Rat | Dhrs11 | Rn.PT.58,9779427 | 145 |
| dehydrogenase/reductase (SDR family) member 11 | Mouse | Dhrs11 | Mm.PT.58.7003308 | 149 |
| hydroxysteroid 11-beta dehydrogenase 1 | Rat | Hsd11b1 | Rn.PT.58.12770485 | 134 |
| hydroxysteroid 11-beta dehydrogenase 1 | Mouse | Hsd11b1 | Mm.PT.58.32968808 | 119 |
| hydroxysteroid (17-beta) dehydrogenase 8 | Rat | Hsd17b8 | Rn.PT.58.12109752 | 92 |
| hydroxysteroid (17-beta) dehydrogenase 8 | Mouse | Hsd17b8 | Mm.PT.58.30674043 | 111 |
| hydroxysteroid (17-beta) dehydrogenase 10 | Rat | Hsd17b10 | Rn.PT.58.9407504 | 130 |
| hydroxysteroid (17-beta) dehydrogenase 10 | Mouse | Hsd17b10 | Mm.PT.58.5744637 | 107 |
| myosin, heavy polypeptide 6, cardiac | Rat | Myh6 | QT00190267 | 127 |
| myosin, heavy polypeptide 7, cardiac | Rat | Myh7 | QT00189504 | 144 |
| natriuretic peptide precursor type A | Rat | Nppa, Anp | QT00366170 | 94 |
| Natriuretic peptide precursor type B | Rat | Nppb, Bnp | Rn.PT.58.5595685.g | 108 |
| TroponinT (cardiac) | Rat | Tnnt | Rn.PT.58.37188705 | 116 |

**Figure S1**: Evaluation by real-time quantitative RT-PCR of the mRNA levels of SDR genes in the LV of rats with acute and severe AR (2 days). Results are reported in arbitrary units (AU) as mean ± SEM (n = 5–6 per group). Levels in sham animals were fixed to 1 (line). *P < 0.05 vs sham. Statistical significance was determined using Student’s t test.

**Figure S2**: Impact of moderate intensity training for a period of 3 months on SDR gene expression in male and female rat cardiac tissue. Results are reported in arbitrary units (AU) as mean ± SEM (n=5-6/gr.). *: p<0.05 vs. sedentary rats of the corresponding sex (line at 1). Statistical significance was determined using Student’s T-test.

**Figure S3**: Impact of gestation on SDR gene expression in female mouse cardiac tissue. Top, heart weight; bottom, SDR gene expression in female rat LV. Results are reported as mean ± SEM (n = 4–8 per group). mRNA levels of non-pregnant rats (NP) were normalized to 1 (line). *P < 0.05 and ****P < 0.0001 vs NP rats. LP, late pregnancy (19 days of gestation); 1PP (24 h post-partum); 4PP, 3–5 days post-partum. Statistical significance was determined using Student’s t test.


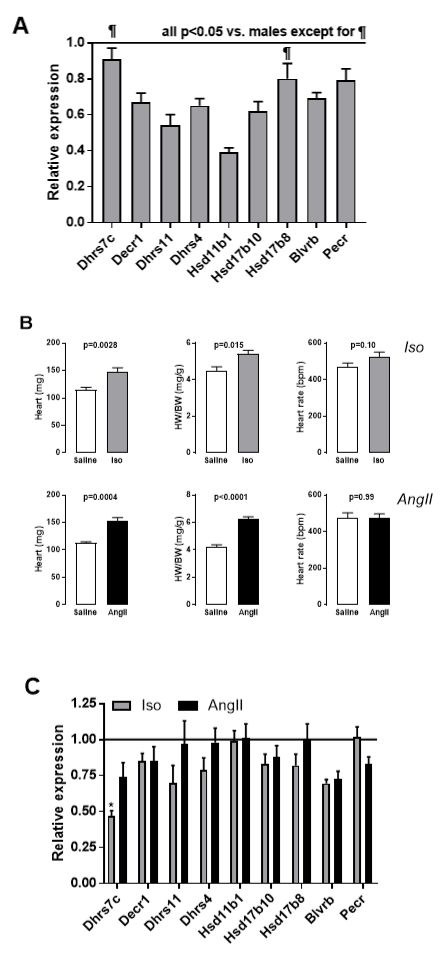


**Figure S4**: Impact of pro-hypertrophic factors on SDR gene expression in female mouse cardiac tissue. (A) SDR gene expression is lower in female mouse LV compared to males. (B) Isoproterenol (Iso; gray bars) or angiotensin II (AngII; black bars) continuous infusion for 14 days results in moderate heart hypertrophy in female mice. (C) Evaluation by real-time quantitative RT-PCR of the LV mRNA levels of the studied SDR genes in female mice. Results are reported in arbitrary units (AU) as mean ± SEM (n = 5–6 per group). mRNA levels of mice receiving vehicle (saline) were normalized to 1 (line). *P < 0.05 vs vehicle-treated mice. Statistical significance was determined using Student’s t test.


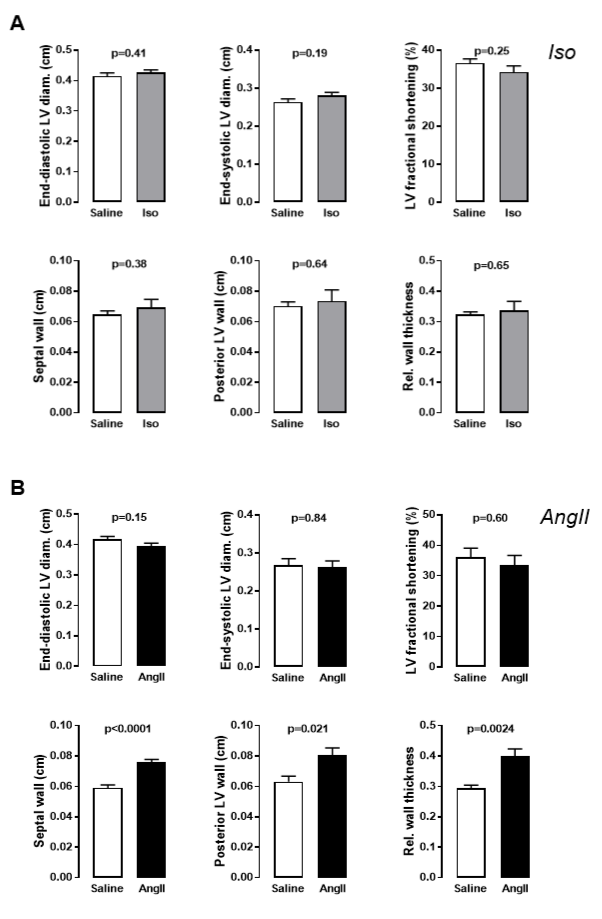


**Figure S5**: Echocardiographic data from female mice treated with continuous infusion of either isoproterenol (Iso) or angiotensin II (AngII) for 14 days. Results are reported as mean ± SEM (n = 5–6 per group). Statistical significance was determined using Student’s t test.
